# A study on waste PCB fibres reinforced concrete with and without silica fume made from electronic waste

**DOI:** 10.1038/s41598-023-50312-z

**Published:** 2023-12-20

**Authors:** M. Vishnu Priyan, R. Annadurai, George Uwadiegwu Alaneme, Durga Prasad Ravella, S. Pradeepkumar, Bamidele Charles Olaiya

**Affiliations:** 1 Department of Civil Engineering, SRM Institute of Science and Technology, Kattankulathur, Chengalpattu, 603203 Tamil Nadu India; 2 Department of Civil, School of Engineering and Applied Sciences, Kampala International University, Kampala, Uganda; 3 Department of Civil Engineering, Michael Okpara University of Agriculture, Umudike, Umudike, Nigeria; 4 Department of Civil Engineering, Chaitanya Bharathi Institute of Technology, Hyderabad, India; 5 Ministry of Environment, Forest and Climate Change, Government of India, New Delhi, India

**Keywords:** Engineering, Materials science

## Abstract

This research goal is to appraise the effect of electronic waste on concrete properties by examining the mechanical properties of concrete reinforced with waste printed circuit boards (PCBs). PCB fibres, each 50 mm long, were mixed in varying proportions (1–5% by weight of cement). Silica fume (SF) was used as a 12% weight replacement for cement to conserve the properties of PCB fibre-reinforced concrete while tumbling cement consumption. Following a 28-day curing period, the fresh and hardened characteristics of PCB fibre-reinforced concrete were juxtaposed with those of conventional concrete. The experimental results led to the conclusion that 5% by weight of cement is the most effective proportion of PCB fibres to include in both PCB fibre-reinforced concrete and silica fume-modified PCB fibre-reinforced concrete. The addition of PCB fibres and silica fume significantly increased the mechanical strength of the concrete, making it suitable for high-strength concrete applications. Based on a similar investigational research design, an artificial neural network model was created, and it played a critical role in predicting the mechanical properties of the concrete. The model produced accurate results, with an R-squared (R^2^) value greater than 0.99.

## Introduction

Electronic waste (e-waste) is a significant issue worldwide, with millions of electronic devices becoming obsolete yearly^1^. Over the next ten years, India is predicted to create 500% more e-waste than it does now, according to the UN Environment Programme (UNEP). Electrical waste equipment (EWE) is being manufactured more widely because of the quick technical improvements in electronic items. This leads to the production of novel products at competitive prices. Valuable metals, man-made chemicals, and hazardous materials that present serious threats to the environment and society are stored at EWE^2^. Advanced countries have included legal and regulatory measures that encourage resource reuse in their waste management programs. On the other hand, a number of developing countries have not made comparable progress in this area^3–5^. In emerging economies, households inefficiently dispose of electronic waste (EWE), resulting in pollution and health hazards. The act of disposing of material waste in neighbouring fields is a common occurrence, leading to the contamination of local sediments, dirt, dust, and vegetation due to inappropriate disposal of electronic trash, resulting in the presence of dangerous substances. This electronic trash consists primarily of screens of LCD (11.9%), computers (18.8%), cell phones (21.3%), and CRT monitors (7%), which are produced by both electrical devices and electronic instruments, manufacturing firms and residents. However, only a scant 10% of this electronic waste is actually being recycled^2–4^. The management of this trash may be achieved by implementing recycling methods using diverse technologies, or alternatively, it can be appropriately dealt with via garbage dumps or incineration processes^5,6^. Nevertheless, inadequate methods of waste disposal and insufficient equipment have the potential to have detrimental effects on both the natural environment and human health. The act of reusing electronic garbage, often referred to as e-waste, may be described as the process of repurposing a product in a manner that deviates from its original intended design and use^2,7^. The implementation of prolonging the product’s life answers, including fix, renovation, and refurbishing, has the potential to significantly prolong the lifetime of goods and make a valuable contribution towards mitigating the issue of electronic waste (e-waste).

Printed Circuit Boards (PCBs) are responsible for approximately 4% of all electronic waste and are electrical interconnections between electronic components^6^. Due to their diverse sizes, materials, and shapes, PCBs are a highly heterogeneous product category^8^. They also contain hazardous substances, such as heavy metals and flame retardants, which require proper treatment to avoid environmental harm. The rapid advancement of technology has led to frequent replacement of electronic devices, leading to an increase in discarded PCBs. The global production of PCBs has a growth rate of 8.7%. In Southeast Asia, the growth rate reached 10.8%, while in China, it reached 14.4%. At present, China is responsible for the production of 40% of the global level^9^. PCBs consist of metals (e.g., aluminium, copper, iron, lead, and tin) and nonmetals (e.g., glass fibres and thermosetting resins). The process of separating metal is an integral component of discarded PCB recycling, which encompasses the utilization of hydrometallurgical, mechanical, and pyrometallurgical techniques^10–12^. However, the challenge of reusing nonmetals from PCBs, which comprise approximately 70% of the material, remains^13–16^.

Presently, non-metallic materials are typically disposed of via incineration or landfilling, both of which can have negative environmental effects. Attempts have been made to recycle nonmetallic materials by incorporating them as fillers in construction materials, and thermoplastic and polyester composites have met with some success. Repurposing nonmetals as fillers for thermoplastic materials may seem ideal, but it runs counter to the current trend of product miniaturization.

PCB waste is a nonbiodegradable material that can persist in the environment for a long time. Nevertheless, the use of this material in construction industries has the potential to mitigate the need for natural resources and mitigate the environmental deterioration resulting from getting rid of it, thus fostering sustainability practices^17,18^. As such, effective waste management strategies are crucial in making the building industry environmentally sustainable, and using waste materials in place of natural resources is a critical consideration. Recycling has several benefits, such as reducing pollution, minimizing waste, and preserving natural resources^19,20^. The current body of research has mostly concentrated on the utilization of PCBs as a viable alternative to natural stone aggregate and cement in the context of concrete construction. However, it is imperative to explore other approaches for the repurposing of this environmentally harmful waste substance^21^. Thus, using PCBs in concrete as fibre strips cut from waste PCBs could be investigated as a viable option. The artificial neural network (ANN) method effectively models different variables in various engineering disciplines^21–23^. This method is a form of artificial intelligence capable of solving intricate and nonlinear problems. The ANN employs software that mimics the biological nervous system of the human brain to process data from different models^24–27^. By using ANN for information processing, highly complex problems can be approached with ease. Environmental and health-related issues have resulted from the increasing accumulation, handling, and disposal of E-waste, particularly Printed Circuit Boards (PCBs), which is the focus of this study^28,29^. The primary objective of this research is to investigate the use of waste PCBs as a replacement for conventional fibres with silica fume in construction practises, with the overarching objective of addressing these issues sustainably. This strategy has the potential to not only reduce costs but also relieve pressure on natural resources and reduce environmental risks. To date, few researchers have attempted to study the properties of concrete containing PCB fibres; hence, experimentally evaluating the mechanical properties and structural strength of concrete reinforced with PCB fibres with and without silica fume is one of the specific aims of this study. In addition, it aims to validate these trial results by relating them to theoretical data for the mechanical characteristics of PCB and SFPCB fibre-reinforced concrete using an artificial neural network (ANN).

## Materials and methods

The cement utilized in this investigation was obtained in the neighbourhood, complied with the BIS 12269-2013^30^ standard, and had a grade of 53 OPC. Cement, with a specific gravity of 3.14, was used. Table 1 summarizes some of the OPC and SF chemical properties. OPC 53 grade cement has a particle size distribution with a mean particle size of 0.0242 mm. The study used M-sand that was purchased locally and had the following specifications: ultimate particle size of 4.75 mm, specific gravity of 2.58, fineness modulus of 2.98, and bulk density of 1672 kg/m^3^. The maximum particle size distribution of the coarse aggregate is 10 mm, the relative density is 2.72, the fineness modulus is 7.11, and the apparent density is 1548 kg/m^3^, which conforms to the BIS 383-2016^31^ standard. Silica fume PCB fibre-reinforced concrete (SFPCB) was produced using silica powder with a relative density of 2.2. In accordance with BIS 456:2000, this experiment created silica fibre-reinforced concrete by substituting 10% of the cement volume^32^. Table 2 displays the M40 mix proportions according to BIS 10262:2009^33^ that are used to cast conventional concrete, silica fume fibre-reinforced concrete (SFPCB), and PCB fibre-reinforced concrete (PCB). The concrete mixture was modified using Conplast SP430, a commercially accessible superplasticizer additive that consists of sulfonated naphthalene polymers, to obtain the desired level of workability. This modification meant that the concrete fulfilled the BIS 9103-1999 specifications^34^. Regular tap water was utilized for both the mixing and curing of the concrete^35^.

**Table 1:** OPC and SF chemical composition.

Chemical composition	Mass (%)	
	OPC	SF
Calcium oxide (CaO)	64.23	0.59
Ferric oxide (Fe_2_O_3_)	3.05	0.98
Sodium Oxide (Na_2_O)	0.15	0.89
Potassium (K_2_O)	0.36	1.09
Silicon dioxide (SiO_2_)	19.34	93.58
Magnesium oxide (MgO)	1.53	1.02
Aluminium oxide (Al_2_O_3_)	4.76	0.55
Sulfur trioxide (SO_3_)	2.01	0.79

**Table 2:** Mix proportions.

Mix specification	Cement	Silica Fume (SF)	M-Sand	Coarse Aggregate	Water	Conplast SP430	WPCB Fibre	WPCB fibre (%)
	(kg/m^3^)							
Conventional concrete	412.5	–	780.39	991.68	164.96	4.125	0	0
PCB	412.5	–	780.39	991.68	164.96	4.125	4.13	1
PCB	412.5	–	780.39	991.68	164.96	4.125	8.25	2
PCB	412.5	–	780.39	991.68	164.96	4.125	12.38	3
PCB	412.5	–	780.39	991.68	164.96	4.125	16.5	4
PCB	412.5	–	780.39	991.68	164.96	4.125	20.63	5
SFPCB	371.25	41.25	780.39	991.68	164.96	4.125	4.13	1
SFPCB	371.25	41.25	780.39	991.68	164.96	4.125	8.25	2
SFPCB	371.25	41.25	780.39	991.68	164.96	4.125	12.38	3
SFPCB	371.25	41.25	780.39	991.68	164.96	4.125	16.5	4
SFPCB	371.25	41.25	780.39	991.68	164.96	4.125	20.63	5

### Printed circuit board fibre

**Figure 1:**
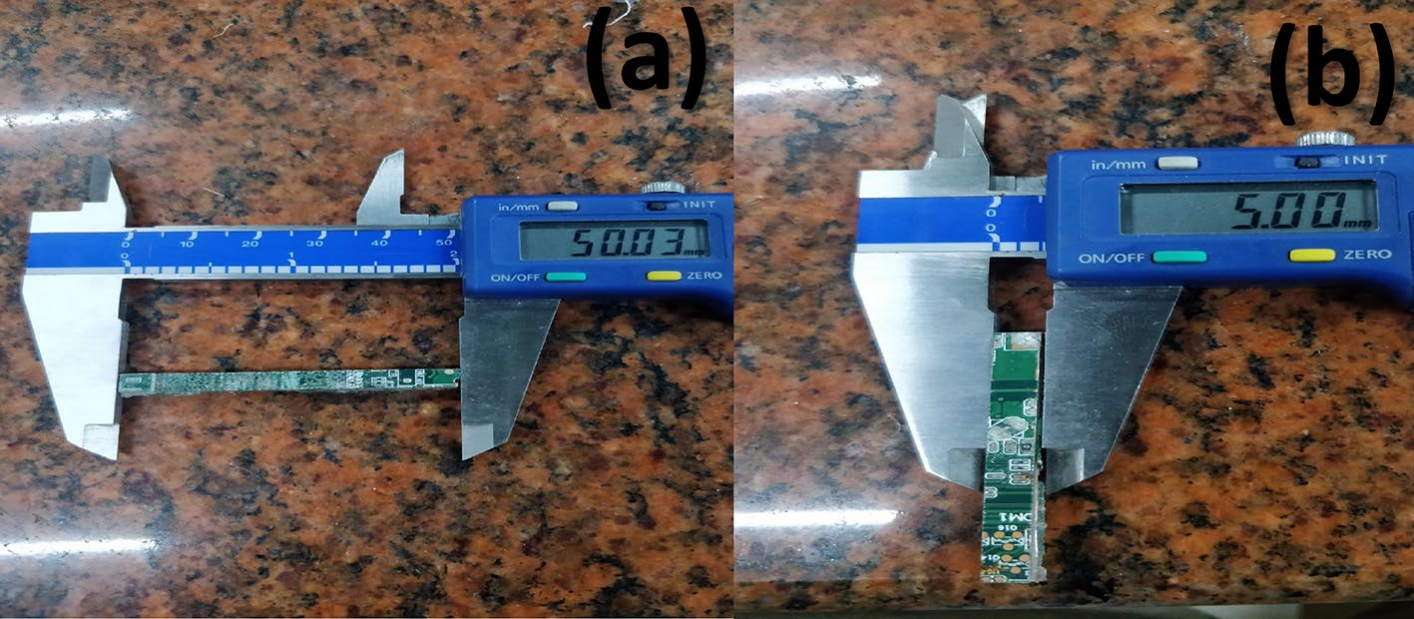
Length and width PCB fibres: AR10 (**a**,**b**).

The FR4-type WPCB used in this study was procured from an electronic waste recycling centre and recovered from obsolete personal PCs. Cleaning discarded Printed Circuit Boards (PCBs) is critical to guarantee proper disposal or reuse. PCB waste cleaning typically entails mechanical disassembly to separate reusable or recyclable components such as electronic components, metal frames, and screws. After removing all metallic parts, the PCB is cut into the desired aspect ratio fibres using a tool and cutter grinder machine^29^. Silica is the primary component of PCBs, with a specific gravity of 2.68 and a PCB fibre tensile strength of 170 N/mm^2^. The measurements of the physical properties of the PCB fibres are summarized in Table 3. This study used PCB fibres with and without silica fume (Fig. 1), as determined by previous research^6^.

**Table 3:** The properties and dimensions of PCB fibres.

Physical properties	Dimension
Length (L) of PCB fibre (mm)	50
Width (W) of PCB fibre (mm)	5
Thickness (t) of PCB fibre (mm)	1.6
Aspect ratio (L/W)	10

### Mix ratio and methods

This experiment examined the effects of adding different percentages of PCB fibres with and without SF aspect ratios AR10 on the mechanical properties of concrete. The mix proportions used in this examination were categorized into three combinations centred on the percentage of added PCB fibre while keeping the cement, coarse aggregates, fine aggregates, silica fume and superplasticizer at constant proportions. A total of 5 unique mixtures were prepared with different percentages of PCB fibre ranging from 1 to 5%, and another five mixes with a combination of silica fume and PCB fibre of 1–5%. The proportion of superplasticizer in all mixes was fixed at 1% of the weight of cement. The concrete mixtures were prepared in a mixer machine, and the mixes were prepared in a conventional manner where fibres were added in parts to achieve uniform dispersions throughout the mix. Fresh concrete tests such as slump were measured following Indian standard BIS 1199-1959^36^. All the samples were demolded the next day and cured in water for a period of 28 days. These samples were tested for compression strength, split tension strength, and flexural strength following BIS 1199-1959 standards^37,38^.

## Experimental results and discussion

### Workability

**Figure 2:**
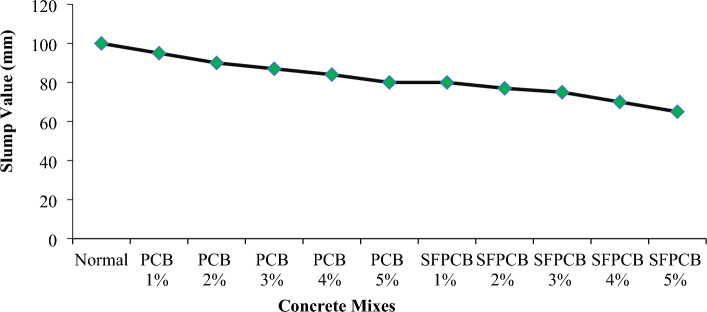
Slump variation in relation to concrete mixes.

The workability of freshly mixed concrete enhanced with PCB fibres was assessed using a slump test. Each mixture was assessed for a slump test three times per batch, and the specimens were cast. Figure 2 depicts the findings of the slump testing. The slump decreased nearly linearly as the PCB fibre content increased. Additionally, the inclusion of silica powder and PCB fibres reduced the workability. In particular, the slump value decreased to 20 mm when 5% PCB fibres were added and to 35 mm when both 5% silica and 5% PCB fibres were added. A greater proportion of PCB fibres in the mixture decreased its workability, indicating an increase in the mixture’s inconsistency. Importantly, all mixtures exhibited a uniform spreading of irregularly positioned detached fibres lacking any indication of fibre clumping. Despite the uniform dispersion of PCB fibre in concrete, the increase in the quantity of fibre caused frictional resistance to the flow of concrete by reducing the slump value. Furthermore, the addition of silica fume increased the specific surface of the particle, causing a reduction in slump^39,40^.

### Properties of WPCB fibre-reinforced hardened concrete

#### Compressive strength

**Figure 3:**
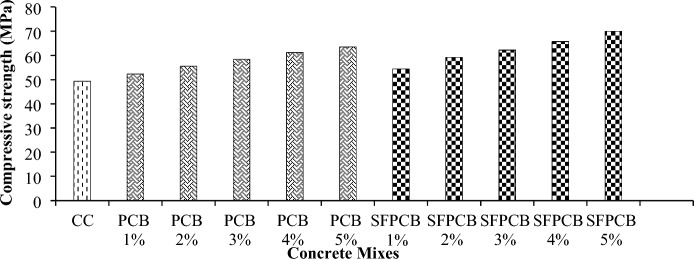
Compressive strength of PCB and SFPCB fibre-reinforced concrete mixes.

The test results of the compressive strength for the PCB and SFPCB fibre-reinforced concrete mixtures after 28 days of curing are depicted in Fig. 3. Following the results of the design mix, the compressive strength of both the PCB and SFPCB fibre-reinforced concrete increased steadily until day 28. The concrete control mix had a compressive strength of 49.35 MPa at 28 days. After 28 days, all PCB fibre-reinforced concrete mixtures exhibited higher compressive strength than the control concrete. Figure 3 illustrates the compressive strengths of each PCB fibre-reinforced concrete mix. Adding PCB fibres at 1%, 2%, and 3% resulted in compressive strength increases of 6.07%, 12.5%, and 18.4%, respectively, compared to the control concrete in the PCB fibre mix. Similarly, the strength in compression increased by 24.11% and 28.77%, including 4% and 5% PCB fibres. The compressive strength of the SFPCB fibre-reinforced concrete prepared with PCB fibres and silica fume followed a similar trend to that of PCB fibres for each of the five mixes after 28 days of curing. Including silica fume and PCB fibres in the concrete has significantly increased its compressive strength compared to PCB fibre and conventional concrete. Adding 1%, 2%, and 3% SFPCB fibre-reinforced concrete mix increased the compressive strength by 10.31%, 20.03%, and 26.1%, respectively. In addition, incorporating fibres at 4% and 5% led to significant increases in compressive strength by 33.2% and 41.6%, respectively. Adding PCB fibres to concrete gradually improves the compressive strength as the percentage of fibres increases. The improved compressive strength can be attributed to a stronger bond between the PCB fibres and the concrete matrix. Incorporating silica powder and PCB fibres into concrete increases stiffness and load-bearing capacity compared to conventional and PCB fibre concrete^41^.

#### Split tensile strength

**Figure 4:**
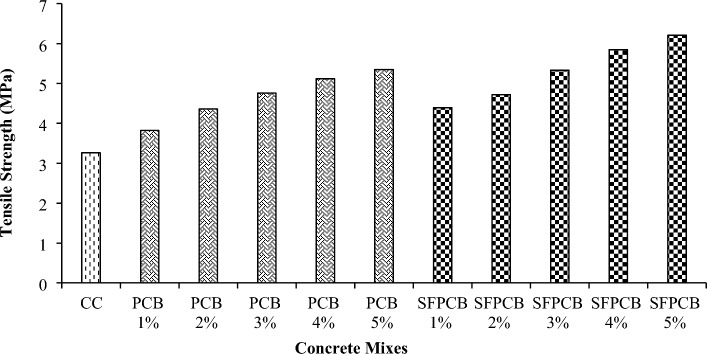
Tensile strength of PCB and SFPCB fibre-reinforced concrete mixes.

A previous study also reported on the tensile strength of PCB fibre-reinforced concrete. Figure 4 illustrates the strength in tension of the PCB fibre and SFPCB fibre-reinforced concrete. Following 28 days of curing, the tensile strength of concrete containing 1%, 2%, 3%, 4%, and 5% PCB fibres and silica fume exceeds that of the regulator concrete. Incorporating a gradual increase of 1% from up to 5% PCB fibres into the mixture resulted in respective increases in tensile strength of 17.17%, 33.74%, 46%, 57%, and 64.11% compared to the control concrete. Figure 4 demonstrates that adding 4% and 5% PCB fibres led to a tensile strength increase of over 50% compared to conventional concrete. The incorporation of 1%, 2%, 3%, 4%, and 5% SFPCB fibres into the concrete resulted in respective increases in tensile strength of 34.66%, 44.78%, 63.49%, 76.38%, and 90.49% compared to control concrete (as depicted in Fig. 4). Adding 3, 4, and 5% SFPCB fibres to the control concrete mixture increased its tensile strength by 40%. Due to the enhanced postcracking effect, the integration of PCB fibres improved the split tensile strength of the concrete. The better tensile strength of an SFPCB mixture compared favorably to that of a PCB fibre, and adding silica powder to the PCB fibre-reinforced concrete increased its split tensile strength due to the mixture’s increased toughness. At the point of ultimate load, the control specimens underwent brittle failure, breaking into two distinct pieces. In contrast, the PCB fibre-reinforced concrete specimens did not exhibit brittle failure and remained intact without separating into two halves^42,43^.

#### Flexural strength

**Figure 5:**
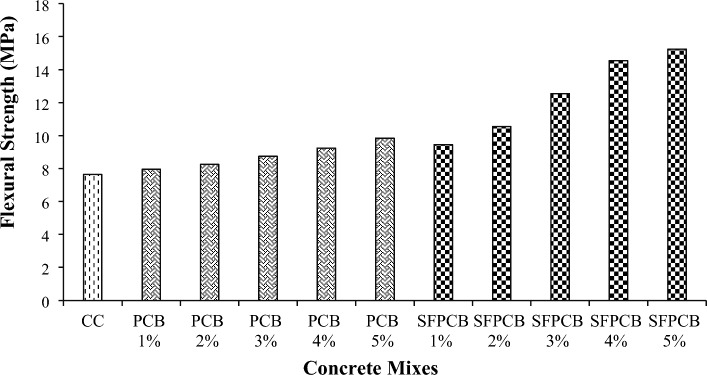
Flexural strength of PCB and SFPCB fibre-reinforced concrete mixes.

The introduction of PCB fibres into the specimen led to a significant enhancement in its load-bearing capability in comparison to the control specimen. The findings of this investigation indicate a correlation between the flexural strength and compressive strength shown by the concrete specimens. Figure 5 illustrates the variations in flexural strength resulting from adding 1, 2, 3, 4, and 5% PCB fibres and SFPCB mix. The flexural behaviour of the mixtures reinforced with PCB fibre and SFPCB increased (Fig. 5). The highest strength was achieved by adding 5% fibre for both the PCB and SFPCB mixes, as shown in Fig. 5, where the strength at 28 days was 9.8 MPa for PCB and 14.25 MPa for SFPCB. Due to the availability of SF and PCB fibres, the flexural strength of all five SFPCB fibre mixes was superior to that of the PCB-based mix^44^.

#### Young’s modulus of PCB fibre-reinforced concrete

**Figure 6:**
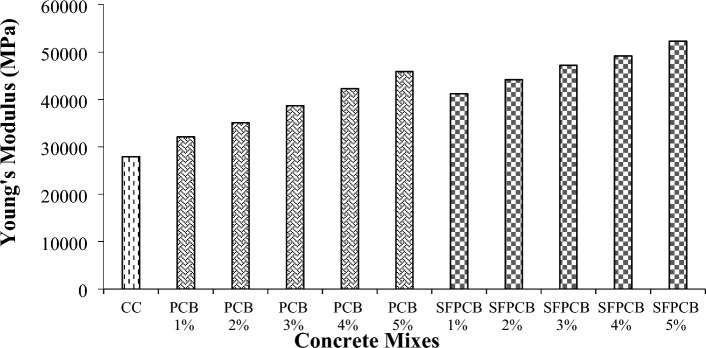
Young’s modulus of the AR10 WPCB fibre-reinforced with and without SF concrete mixes.

The Young’s modulus of the concrete mixes with PCB and SFPCB fibre reinforced concrete is shown in Fig. 6. This study used 300 mm in height and 150 mm in diameter concrete cylinders. Figure 6 depicts the Young’s PCB and SFPCB fibre-reinforced concrete modulus measured after 28 days as per ASTM C469-2022^45^. The increase in Young’s modulus is 87% or more than that of the control concretes. Additionally, it is worth noting that the Young’s modulus of WPCB fibre-reinforced concrete is much greater than that of the control specimen. This holds true for both the mixes reinforced with PCB and SFPCB in the concrete composition. The Young’s modulus value of the SFPCB fibre-reinforced concrete combination was greater than that of the PCB fibre-reinforced concrete mix. The maximum values of Young’s modulus were attained for the concrete mixed with 5% PCB and SFPCB fibres, which were 64.5% and 87%, respectively. The higher Young’s modulus is because of the improved bonding between the PCB fibre and the concrete matrix^46^.

## ANN results

**Figure 7:**
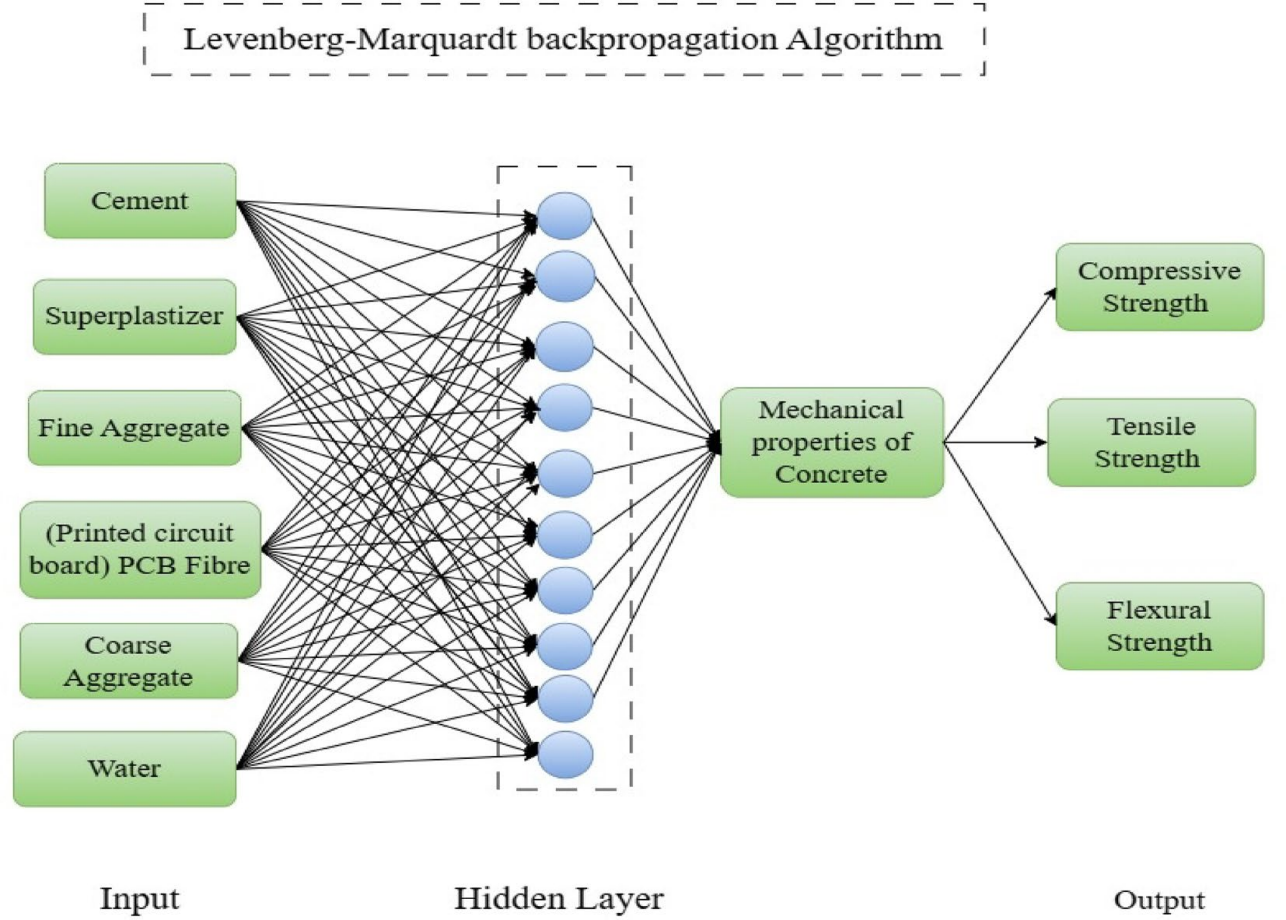
ANN architecture of PCB Fibre concrete.

This study considers a single concealed layer for the ANN architecture. Therefore, a six-layer network is employed and depicted in Fig. 7. The first and second layers consist of six input and seven hidden neurons, respectively. The third layer consists of the mechanical properties of the PCB and SFPCB fibre-reinforced concrete as the output layer^47^.

**Figure 8:**
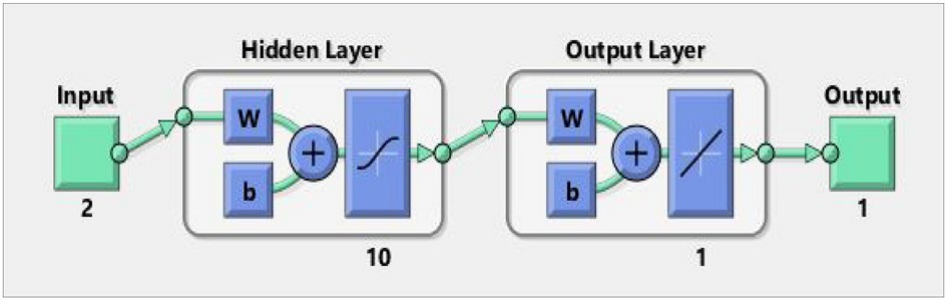
ANN structure model in MATLAB.

The ANN model incorporates empirically determined mechanical characteristics of PCB fibre concrete for the purpose of validation and then discusses these mechanical features. The mean square error of the network shown in Fig. 8 characterizes the attributes of PCB fibre concrete, exhibiting an initial high value that gradually diminishes towards lower values.

**Figure 9:**
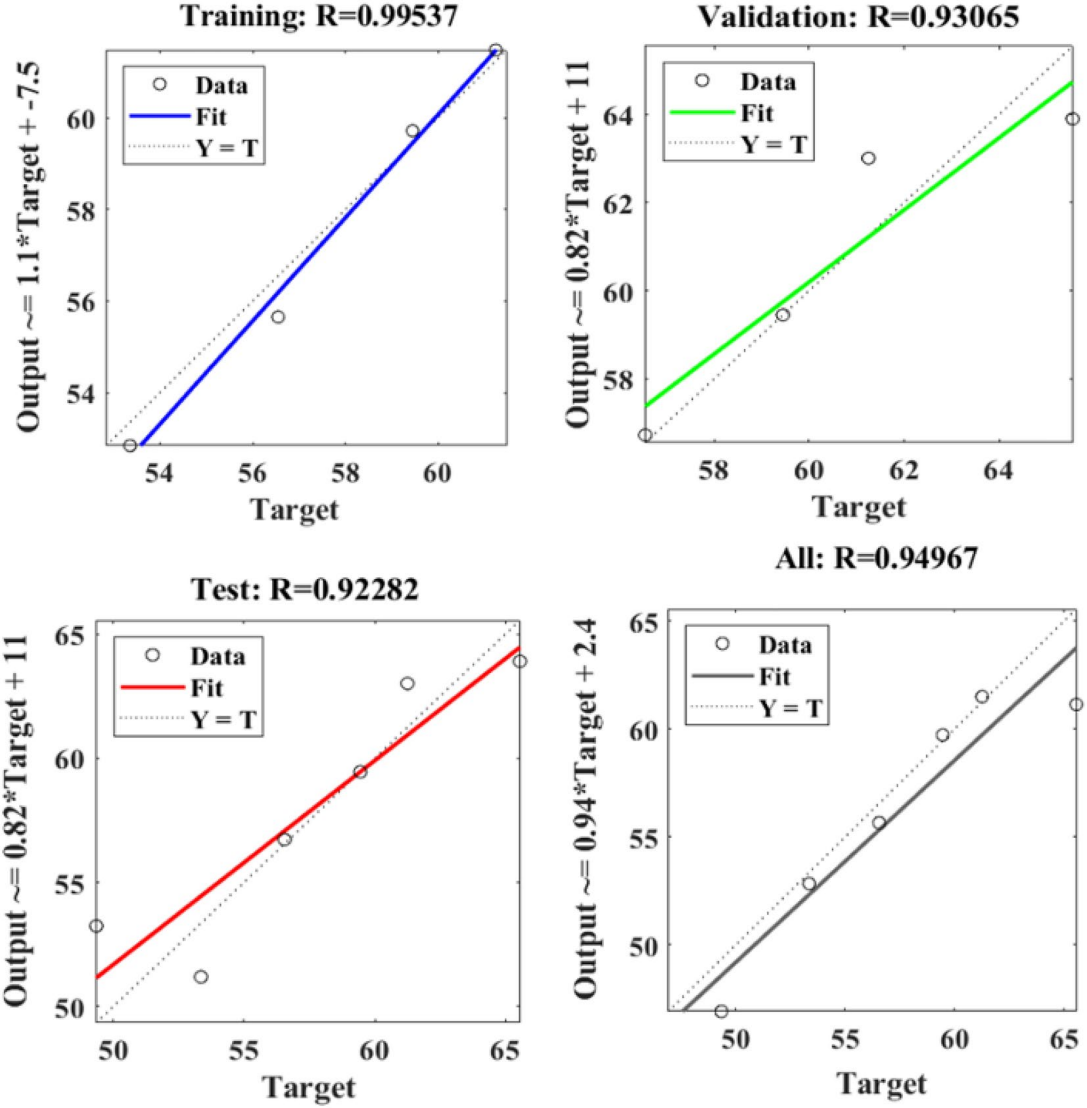
ANN regression plot of compressive strength for PCB fibres.

**Figure 10:**
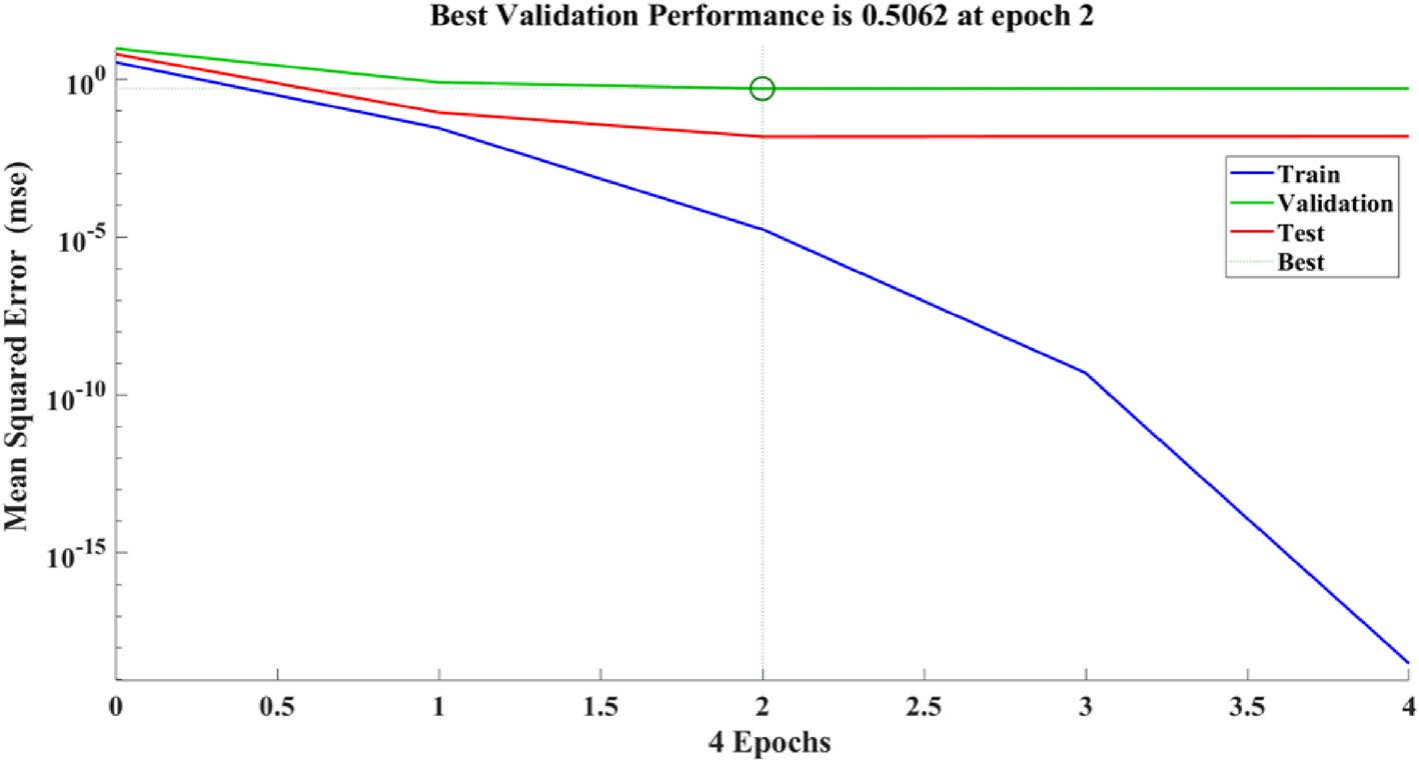
Performance plot of the compressive strength of PCB fibre-reinforced concrete.

**Figure 11:**
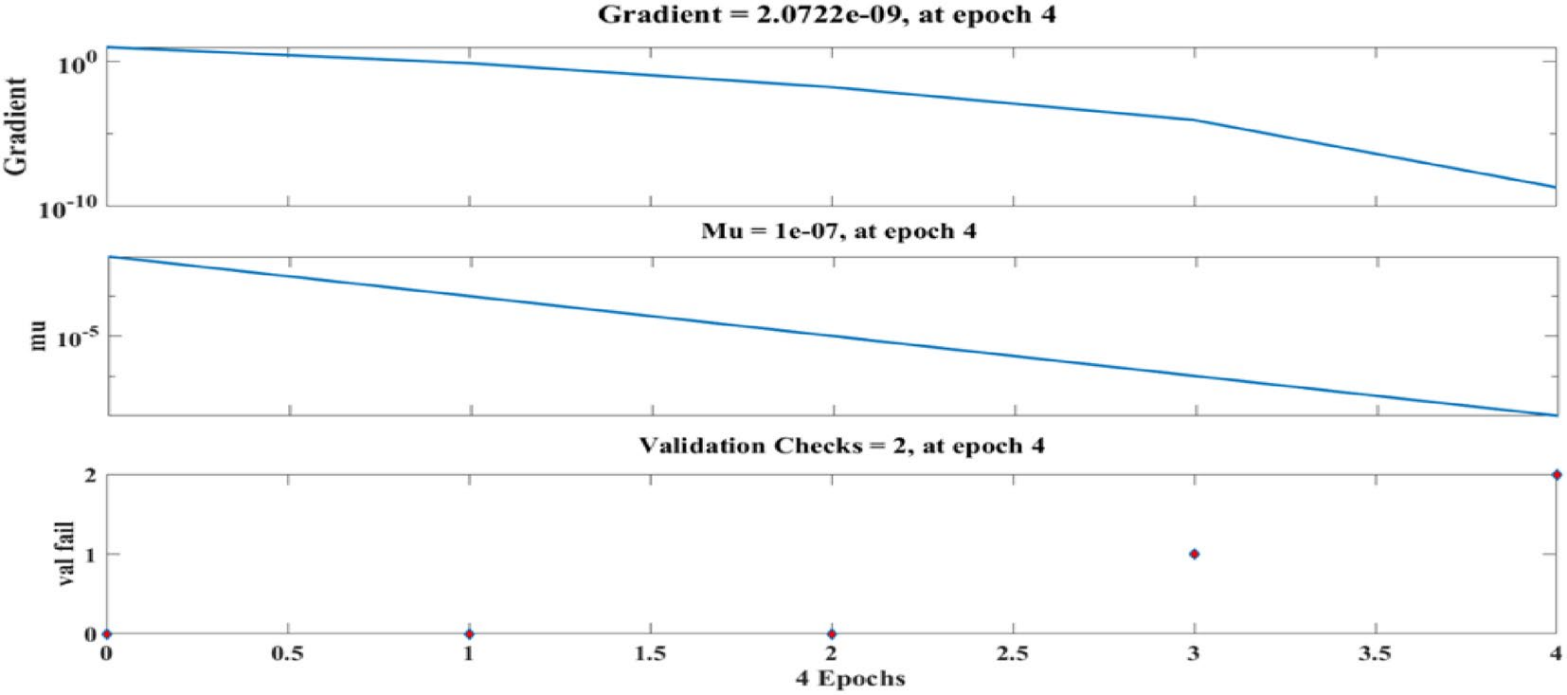
Fit plot of the compressive strength of PCB fibre-reinforced concrete.

Figure 9 depicts the regression plots of the proposed artificial neural network (ANN) model for the compressive strength of PCB fibre-reinforced concrete. The diagram illustrates that the R values utilized for training, validation, and testing of the model exceed 0.99537, 0.93065, and 0.92282, respectively. The network mean squared error (MSE) is illustrated in Figs. 10 and 11, demonstrating a declining pattern, as anticipated for a proficiently trained artificial neural network (ANN). This trend serves as a reliable indicator of the network’s learning progression. Due to the random segmentation of the ten hidden layers and target vectors into three sets, the plotted graph contains three lines. After successfully operating on the training set, training ceases, thereby avoiding the problem of overfitting^48,49^.

**Figure 12:**
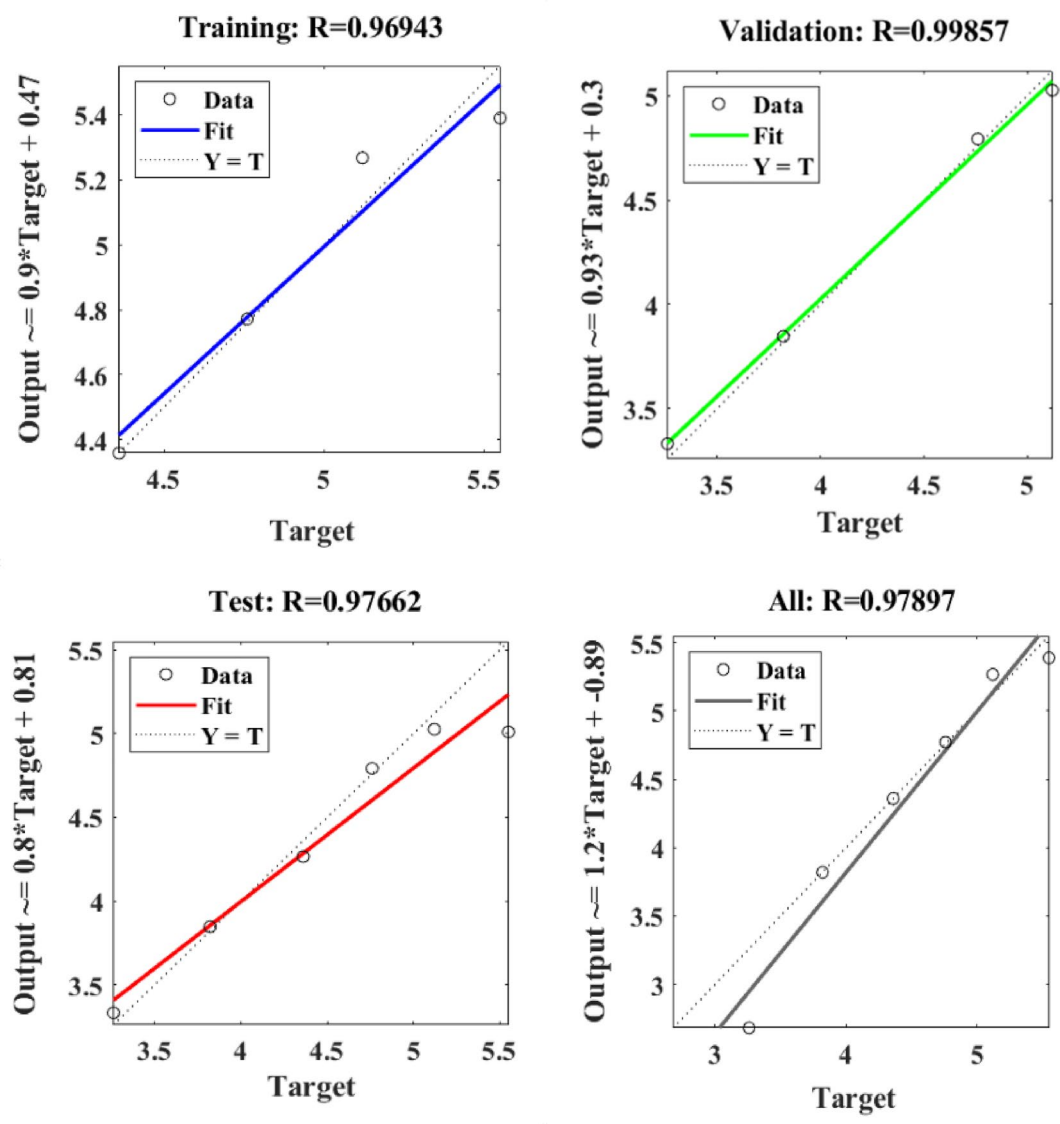
ANN regression plot of tensile strength for PCB fibres.

**Figure 13:**
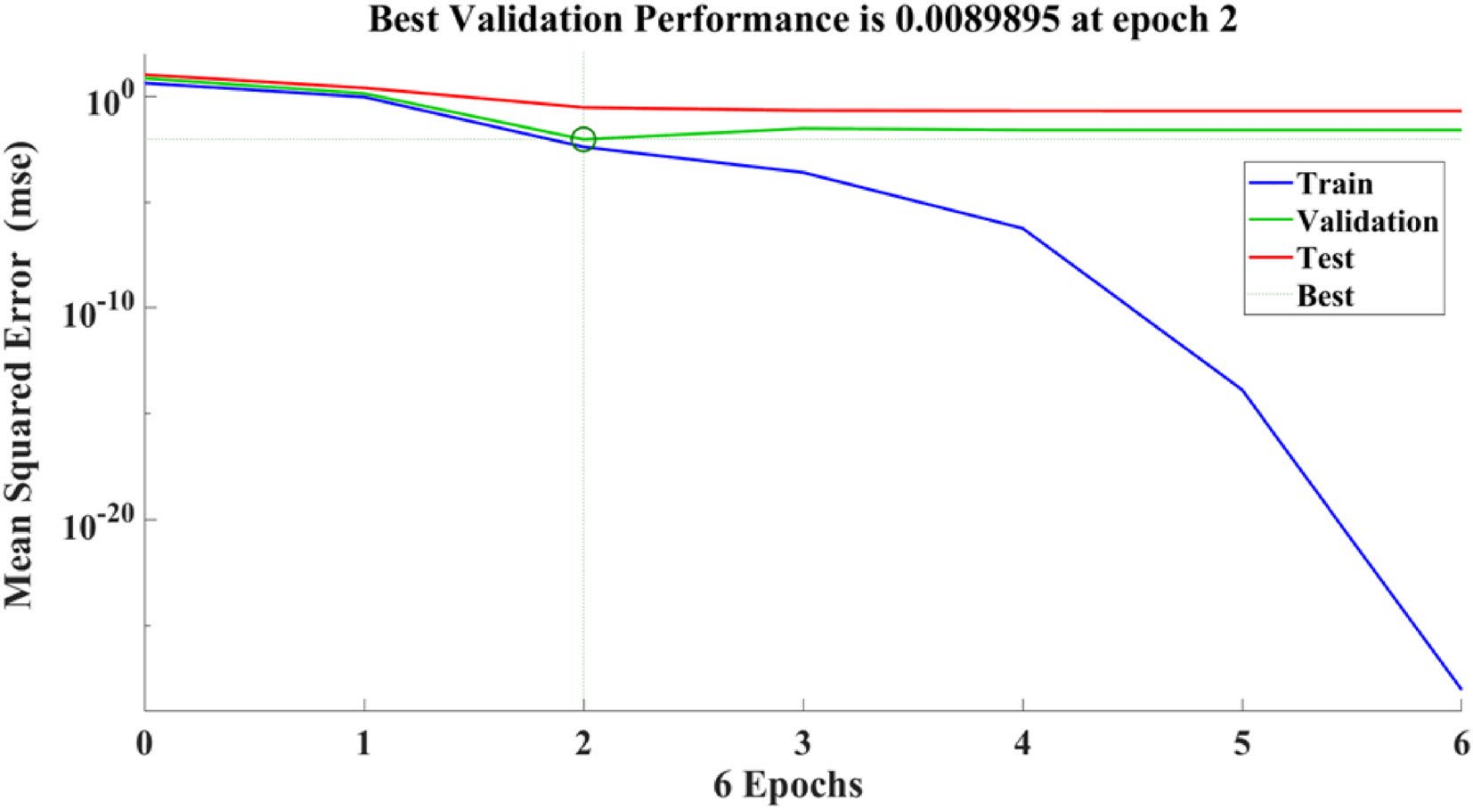
Performance plot of the tensile strength of PCB fibre-reinforced concrete.

**Figure 14:**
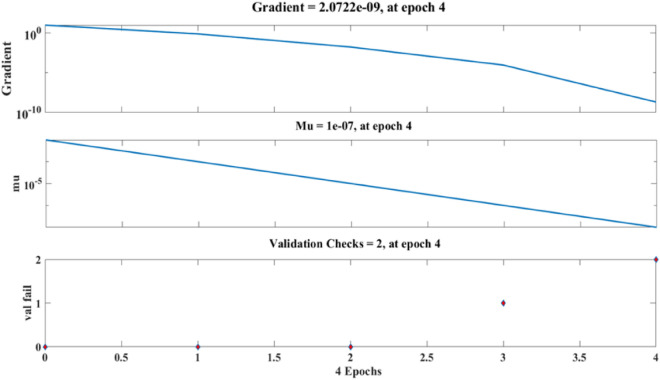
The fit curve for the tensile strength of PCB fibre-reinforced concrete.

Figure 12 depicts the regression plots of the proposed artificial neural network (ANN) model for the tensile strength of PCB fibre-reinforced concrete. The diagram illustrates that the R values employed for model training, validation, and testing are all in excess of 0.96943, 0.99857, and 0.97662, respectively. The network mean squared error (MSE) is illustrated in Figs. 13 and 14. The observed trend in the MSE is consistent with the anticipated behaviour of a proficiently trained artificial neural network (ANN) and serves as a reliable metric for evaluating the network’s learning performance. The figure displays three lines as a result of the stochastic partitioning of the ten concealed layers and objective vectors into three distinct sets. After successfully operating on the training set, training ceases, thereby avoiding the problem of overfitting^50,51^.

**Figure 15:**
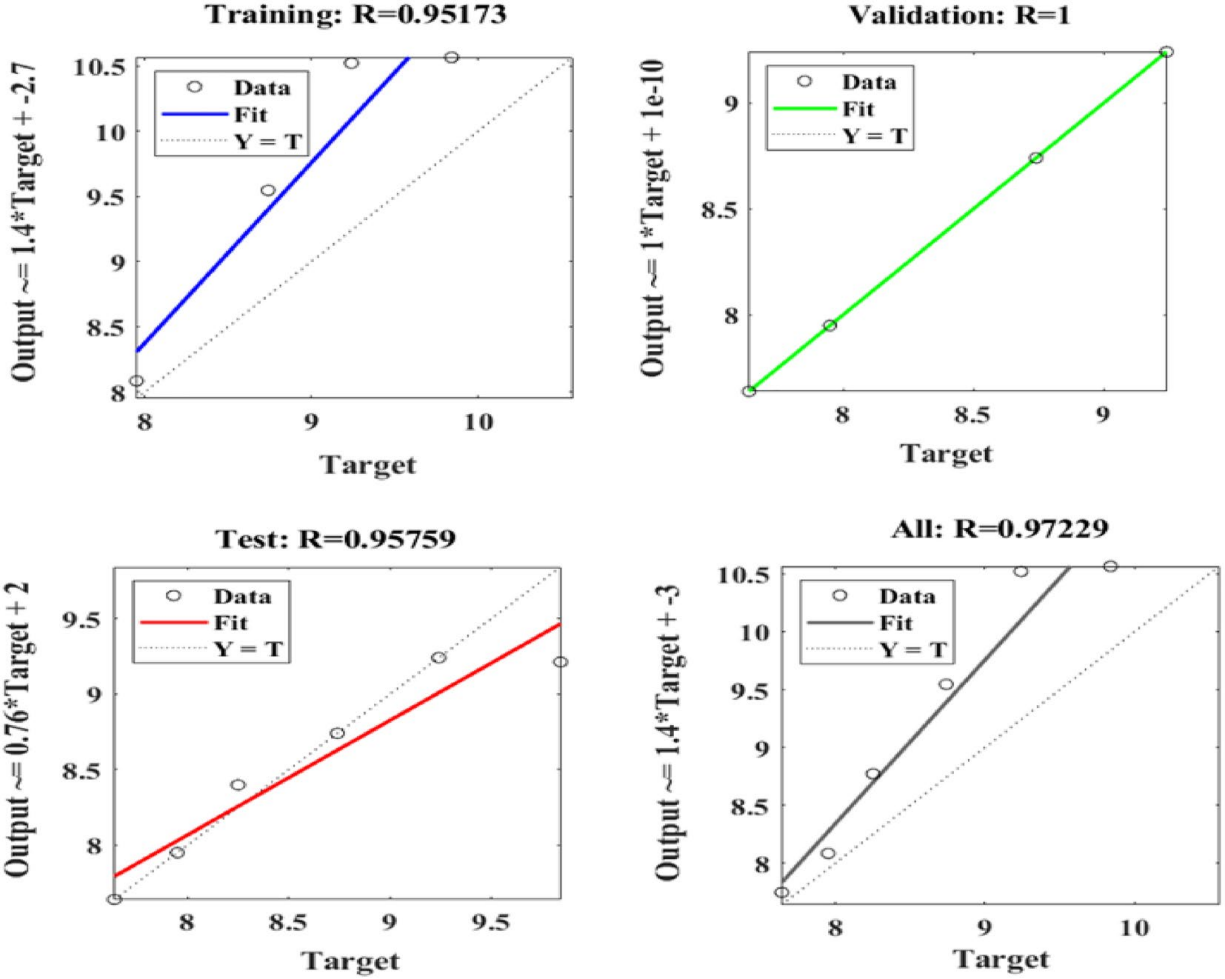
ANN regression plot of the flexural strength of PCB fibre-reinforced concrete.

**Figure 16:**
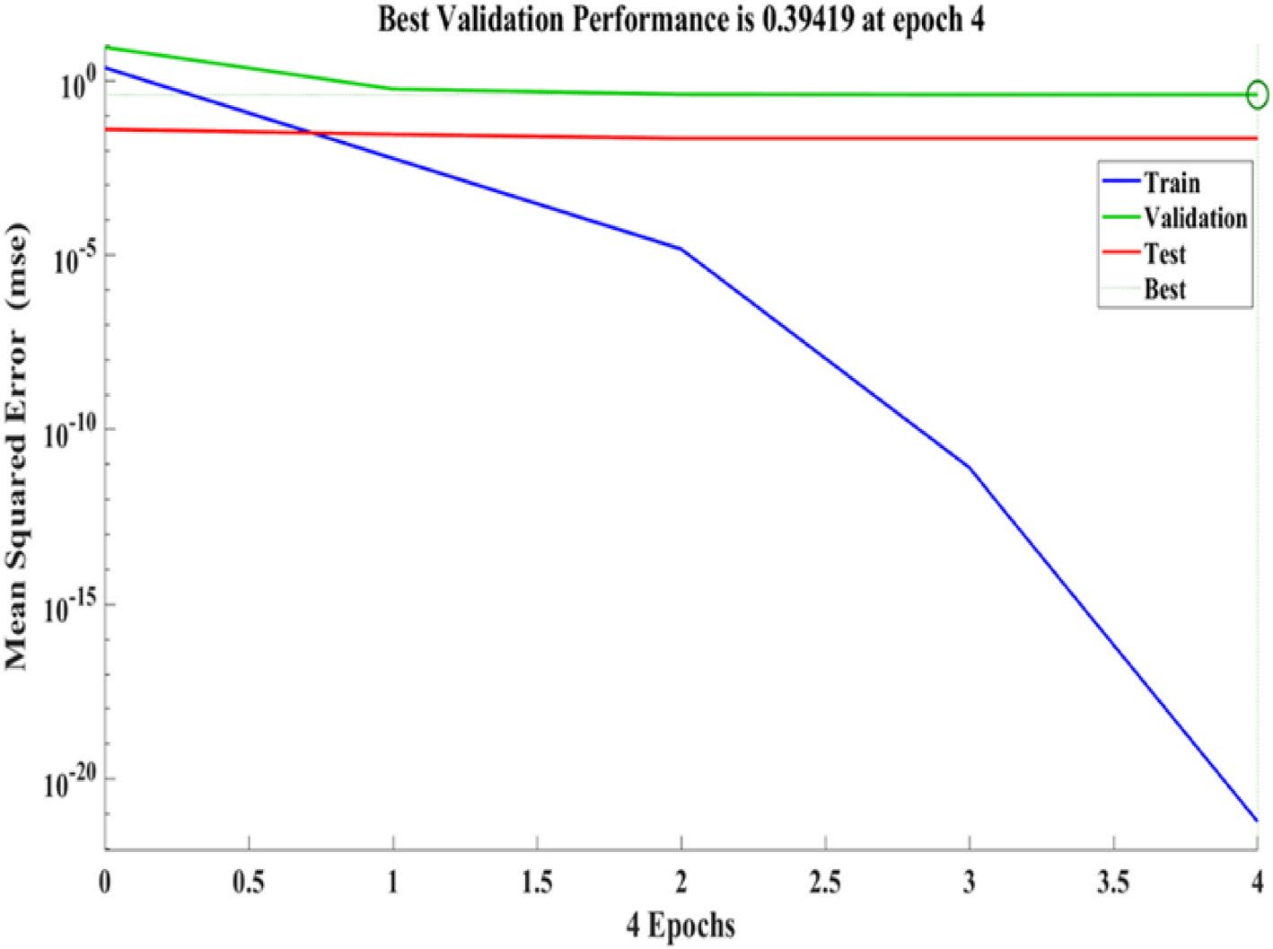
Performance plot of the flexural strength of PCB fibre-reinforced concrete.

**Figure 17:**
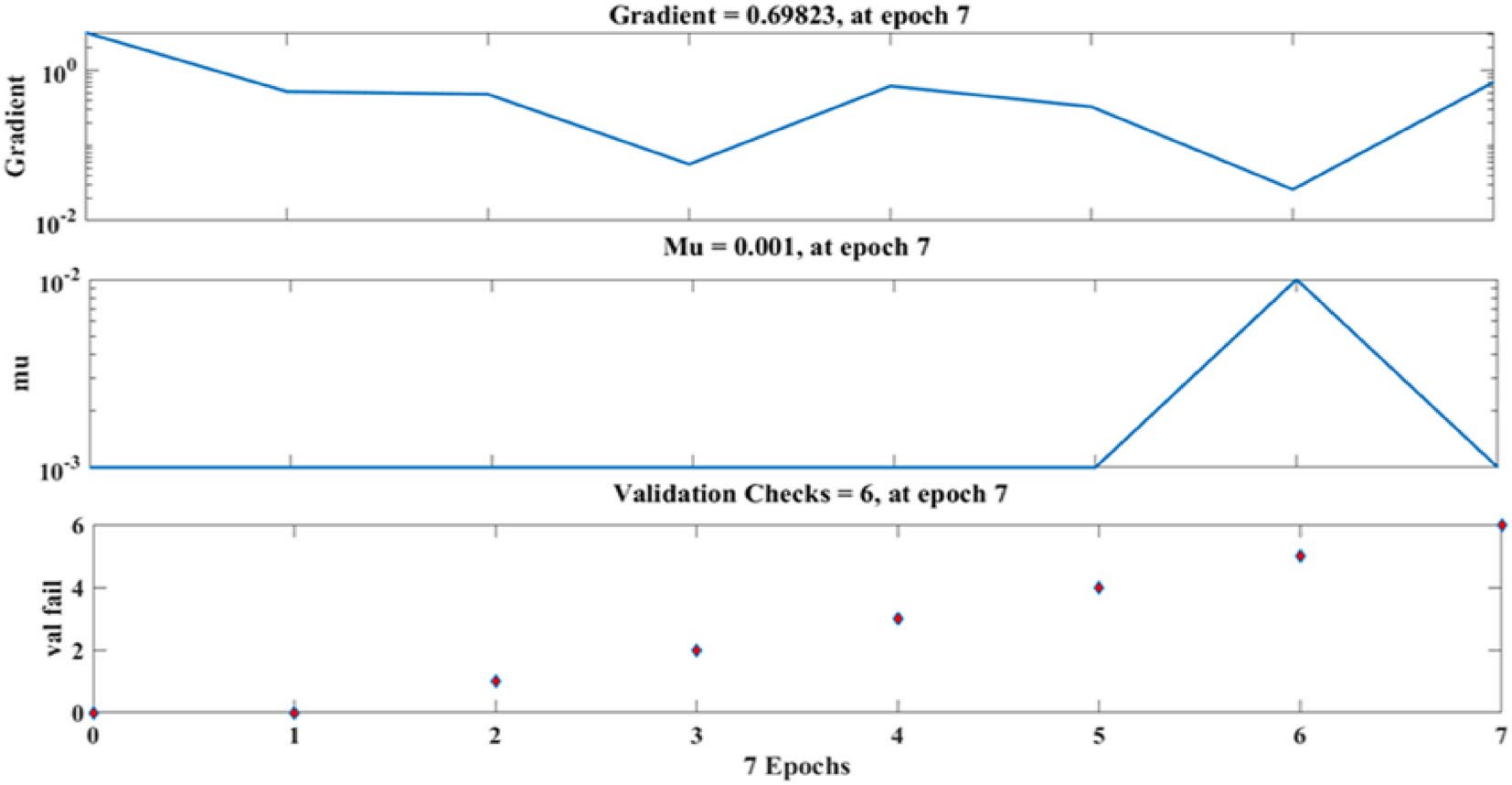
Fit plot of the flexural strength of PCB fibre-reinforced concrete.

Figure 15 depicts the regression plots of the proposed artificial neural network (ANN) model for the flexural strength of PCB fibre-reinforced concrete. The R values used to train, validate, and test the model are all greater than 0.95713, 1, and 0.95759, respectively, as shown in the diagram. The network mean squared error (MSE) is illustrated in Figs. 16 and 17. The observed trend is a decrease, which aligns with the anticipated behaviour of a proficiently trained ANN. Additionally, the MSE serves as a reliable metric for assessing the network’s learning progression. The figure depicted in the plot exhibits three distinct lines, which can be attributed to the arbitrary partitioning of the ten concealed layers and objective vectors into three distinct sets. After successfully operating on the training set, training ceases, thereby avoiding the problem of overfitting^52^.

**Figure 18:**
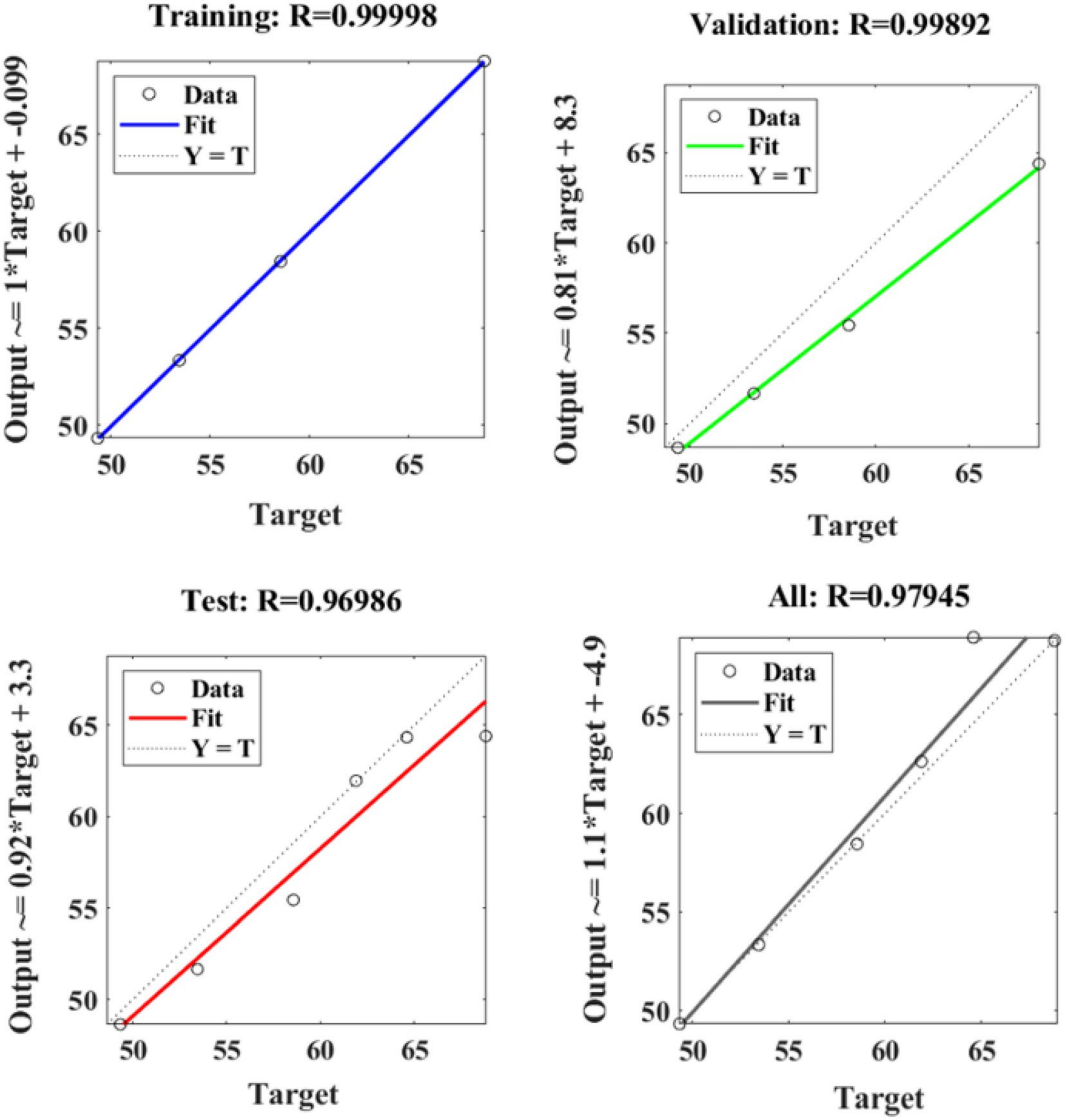
ANN regression plot of the compressive strength of SFPCB fibre-reinforced concrete.

**Figure 19:**
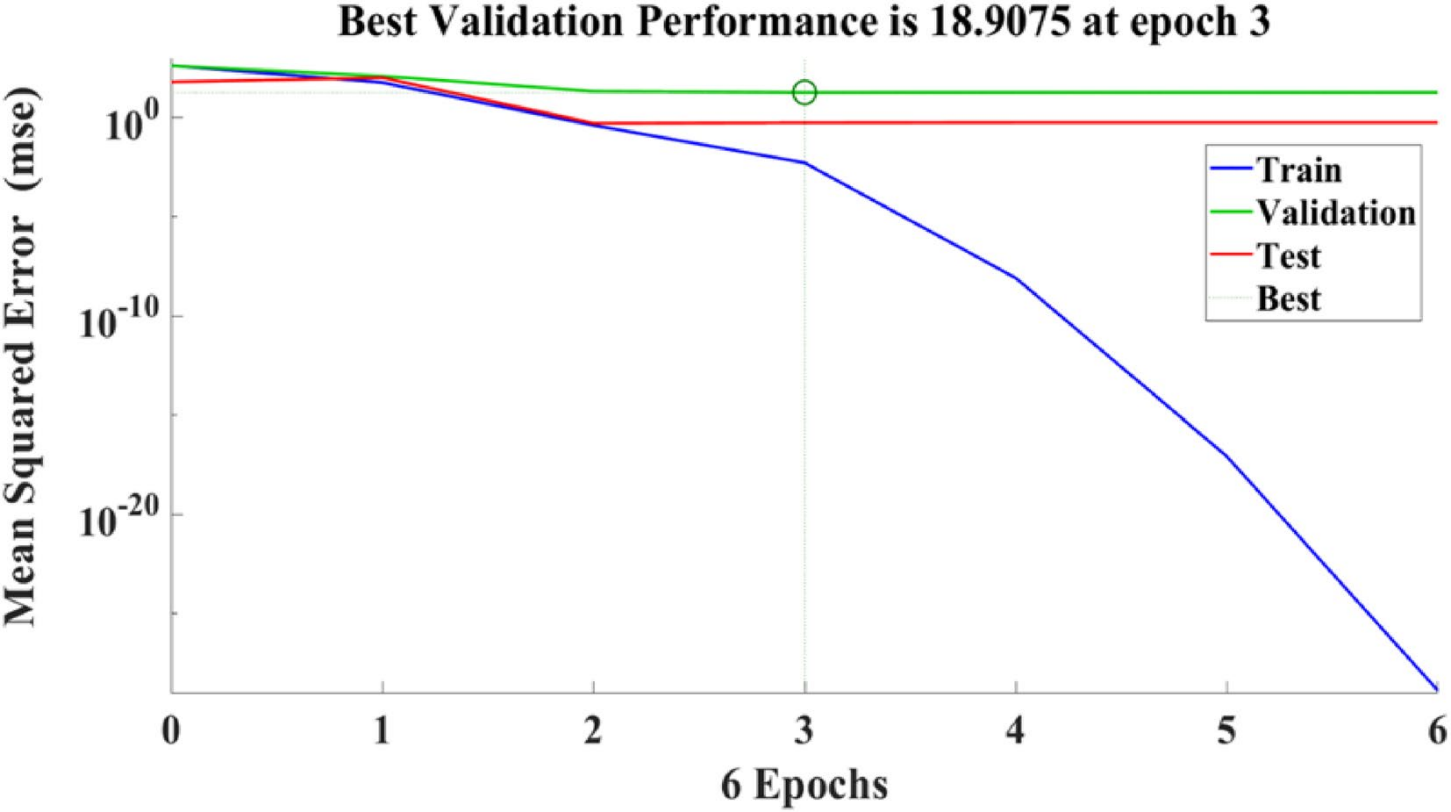
Performance plot of the compressive strength of SFPCB fibre-reinforced concrete.

**Figure 20:**
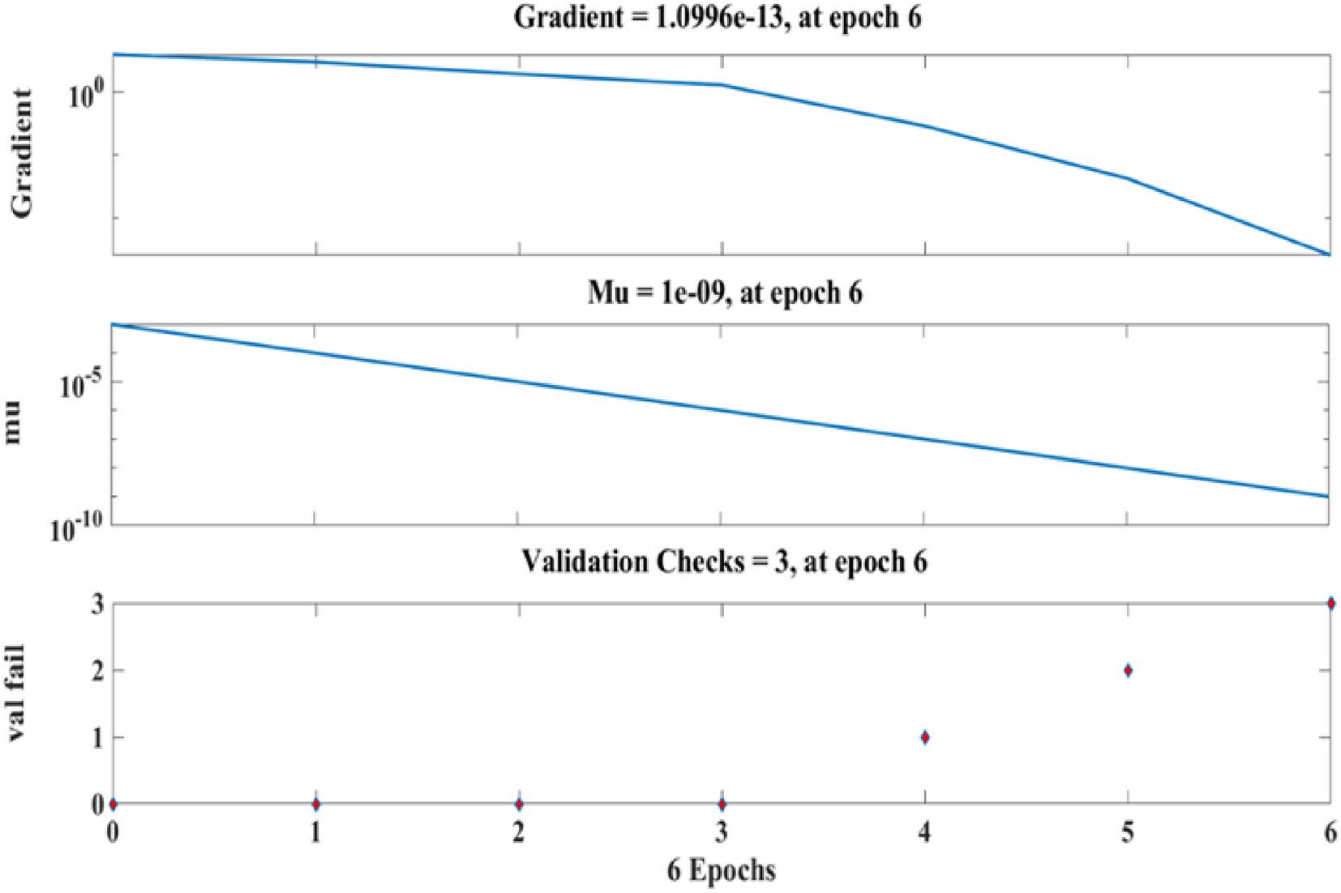
Fit plot of compressive strength of SFPCB fibre-reinforced concrete.

Figure 18 depicts the regression plots of the SFPCB fibre-reinforced concrete compressive strength as proposed by the ANN model. The diagram displays R values for model training, validation, and testing, which are all above 0.99998, 0.99892, and 0.96986, respectively. The network mean squared error (MSE) is illustrated in Figs. 19 and 20. The observed trend is a decrease, which aligns with the anticipated behaviour of a proficiently trained ANN. Additionally, the network’s learning process can be effectively monitored by examining the MSE. The figure presented in the analysis depicts three distinct lines as a result of the stochastic partitioning of the ten concealed layers and objective vectors into three distinct subsets. After successfully operating on the training set, training ceases, thereby avoiding the problem of overfitting^53,54^.

**Figure 21:**
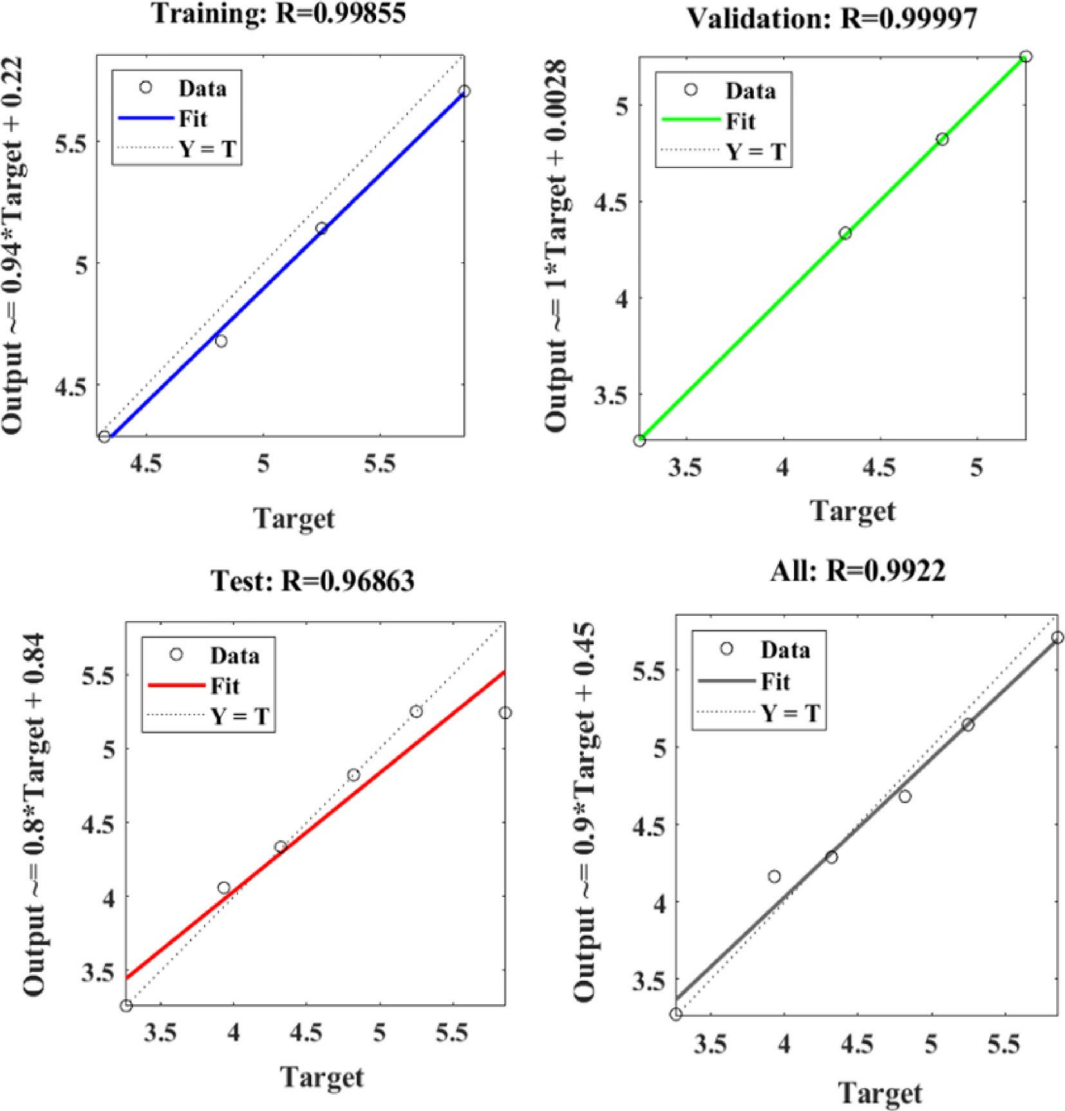
ANN regression plot for the tensile strength of SFPCB fibre-reinforced concrete.

**Figure 22:**
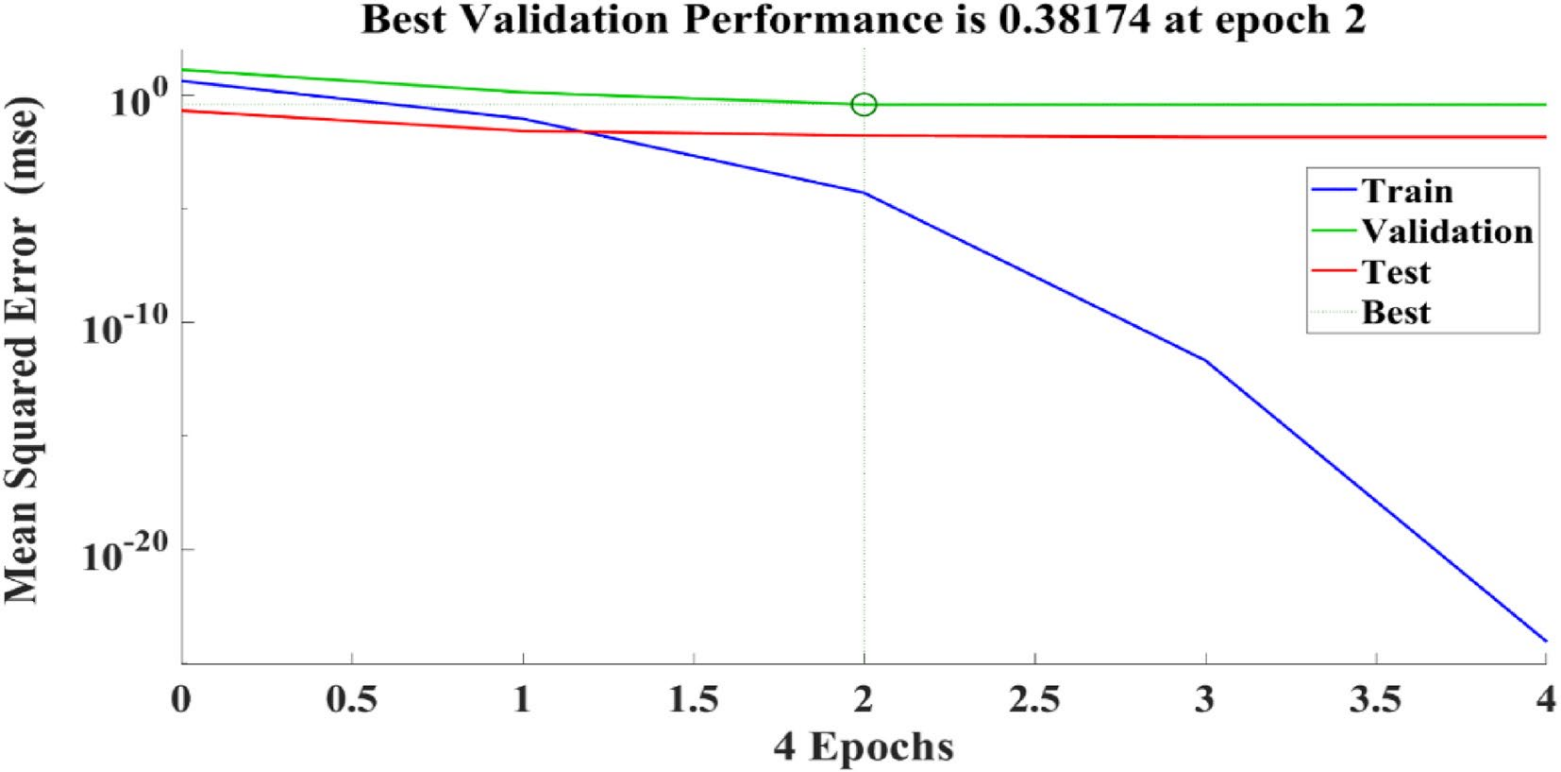
Performance plot for tensile strength of SFPCB fibre-reinforced concrete.

**Figure 23:**
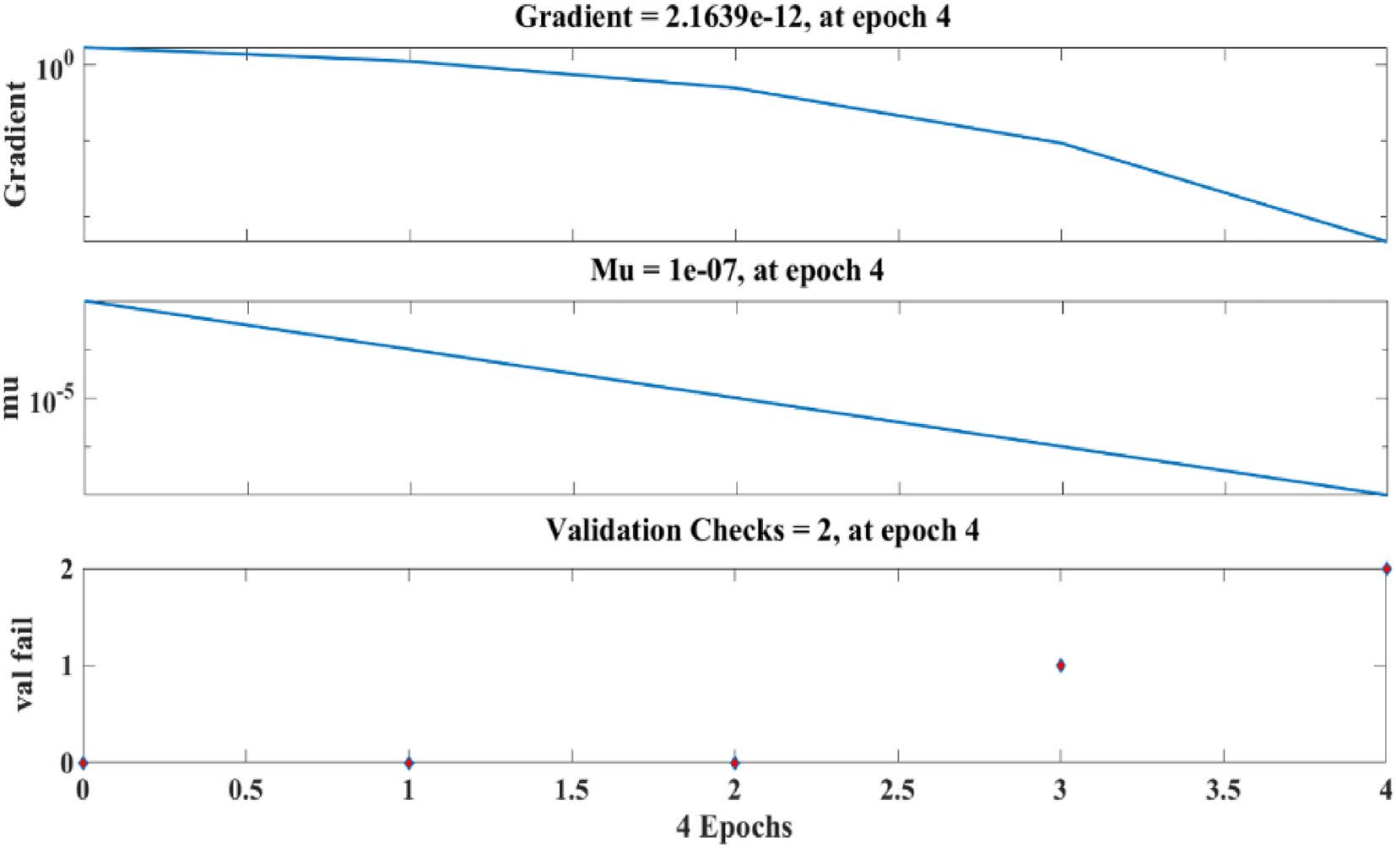
Fit plot for the tensile strength of SFPCB fibre-reinforced concrete.

Figure 21 depicts the regression plots of the proposed artificial neural network (ANN) model for the tensile strength of SFPCB fibre-reinforced concrete. The diagram displays R values for model training, validation, and testing, which are all above 0.99855, 0.99997, and 0.96863, respectively. The network mean squared error (MSE) is illustrated in Figs. 22 and 23, demonstrating a decline that aligns with the anticipated behaviour of a proficiently trained artificial neural network (ANN). This metric serves as a valuable gauge of the network’s learning progression. Due to the random segmentation of the ten hidden layers and target vectors into three sets, the plotted graph contains three lines. After successfully operating on the training set, training ceases, thereby avoiding the problem of overfitting^55,56^.

**Figure 24:**
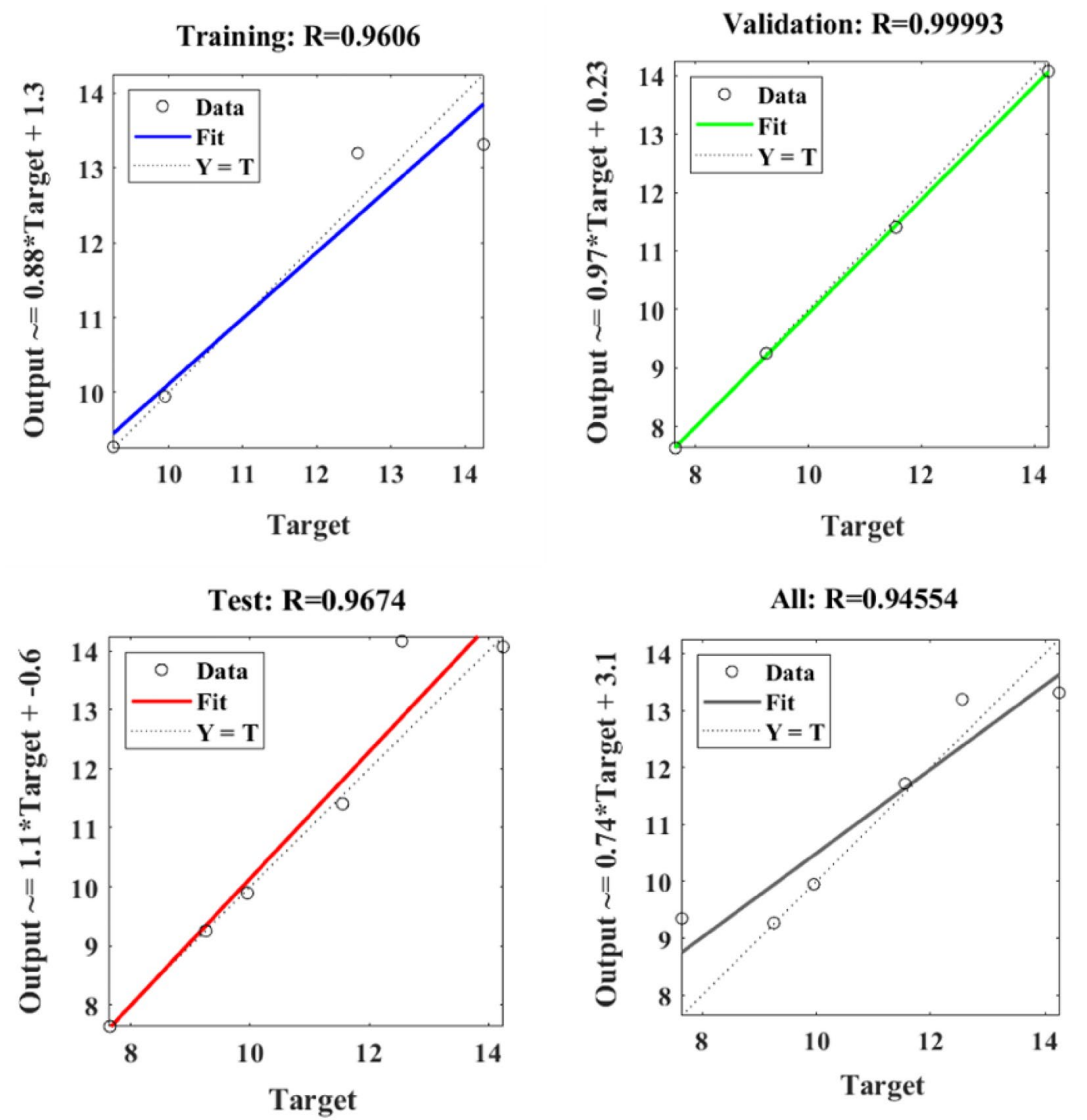
ANN regression plot for the flexural strength of SFPCB fibre-reinforced concrete.

**Figure 25:**
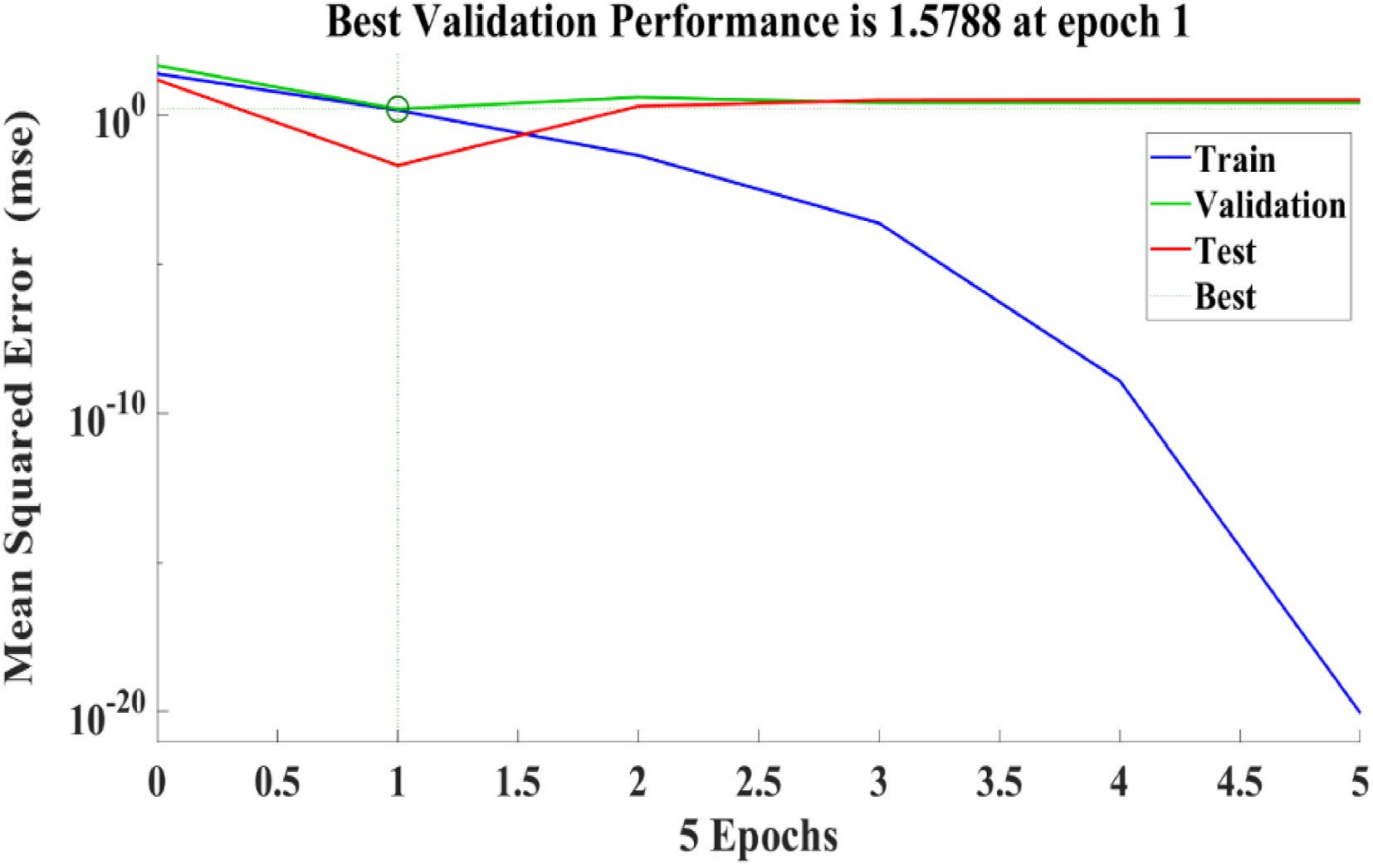
Performance plot for flexural strength of SFPCB fibre-reinforced concrete.

**Figure 26:**
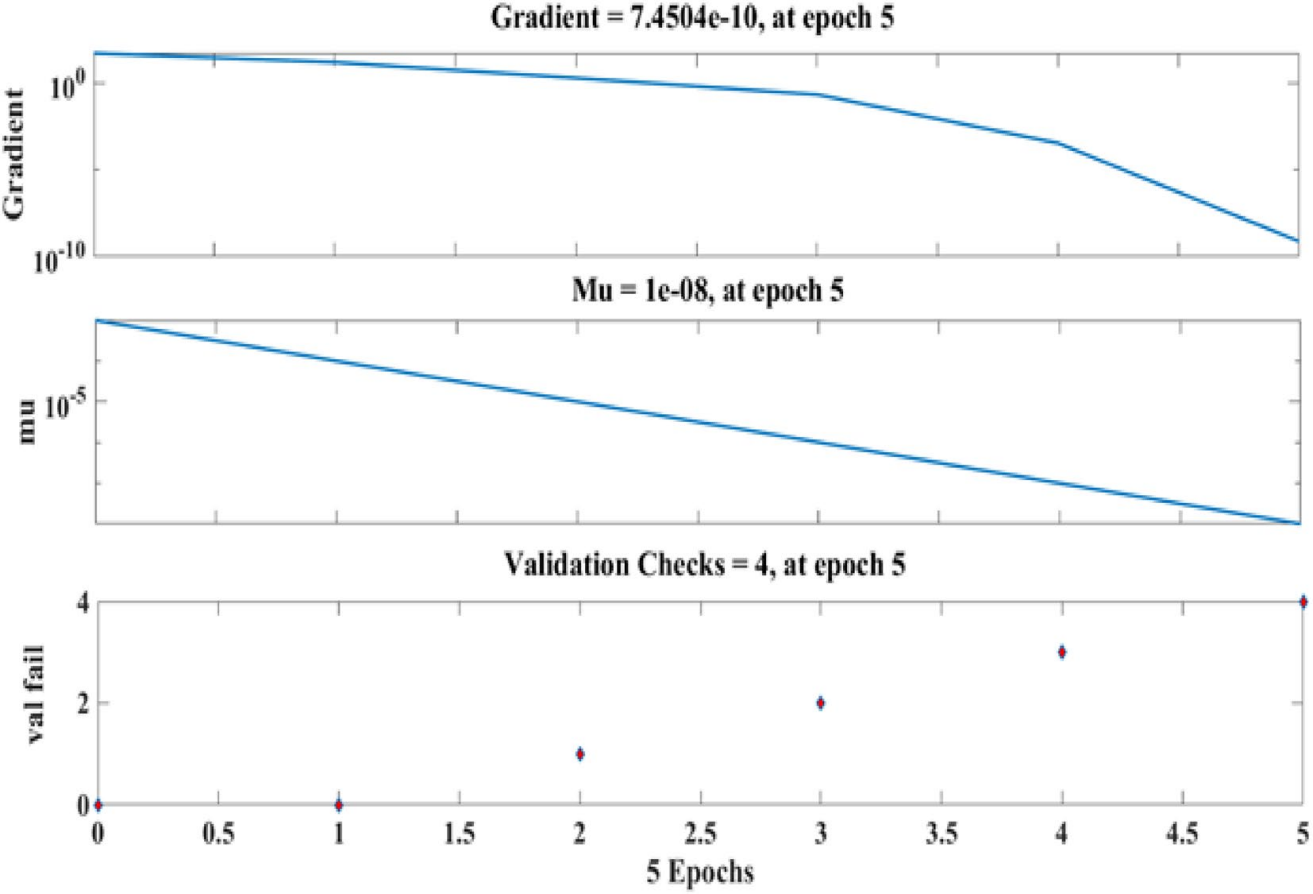
Fit plot for the flexural strength of SFPCB fibre-reinforced concrete.

Figure 24 depicts the regression plots of the proposed ANN model for the flexural strength of SFPCB fibre-reinforced concrete. The diagram illustrates that the R values utilized for training, validation, and testing of the model exceed 0.9606, 0.99993, and 0.9674, respectively. The network mean squared error (MSE) is illustrated in Figs. 25 and 26. The observed trend in the MSE is consistent with the anticipated behaviour of a proficiently trained artificial neural network (ANN) and serves as a valuable metric for assessing the network’s learning progression. Due to the random segmentation of the ten hidden layers and target vectors into three sets, the plotted graph contains three lines. After successfully operating on the training set, training ceases, thereby avoiding the problem of overfitting^52,57^.

The R correlation coefficients for the compressive, tensile, and flexural strengths of both the PCB and SFPCB fibre-reinforced concrete were calculated using ANN analysis to be 0.94967, 0.97897, 097,229, 0.97945, 0.9922 and 0.94554, respectively. Because the correlation coefficient is close to 1 for all models, the ANN model was appropriate^58,59^.

## Conclusions

Extensive research was conducted to thoroughly examine the mechanical properties of both PCBs and SFPCBs. The influence of PCB fibres and silica fume on the properties of fresh and hardened concrete was empirically established, and these findings were subsequently verified through an artificial neural network (ANN)-based statistical model. The study led to the derivation of key insights and conclusions:

1. In comparison to the control concrete, the inclusion of silica fume (SF) and a higher fibre (5%) content in the composite resulted in a notable improvement in compressive strength. Specifically, the compressive strength increased by 32.8% for PCB and by 40.8% for SFPCB. The increase in the compressive strength can be attributed to the increase in the bonding between the PCB fibre and cement matrix and the increase in the matrix strength due to the presence of silica fume.
2. The tensile strength of PCB fibre 5% and SFPCB 5% increased by 70% and 80.1%, respectively, in comparison to conventional concrete. The trend closely matched the findings of compressive strength, which was due to the presence of SF and PCB fibres. The flexural strength of the PCB and SFPCB fibre-reinforced concrete exhibited a similar pattern as that observed in the results of the compressive and tensile strength tests.
3. The R correlation coefficients for the compressive, tensile, and flexural strengths of the PCB and SFPCB fibre-reinforced concrete were calculated using ANN analysis to be 0.94967, 0.97897, 0.97229, 0.97945, 0.9922 and 0.94554, respectively, and the values tend to prove that the use of such a numerical model can be beneficial in predicting the mechanical characteristics of concrete.
4. This study found that the concrete sample’s mechanical properties improved directly as a proportion of added SF and PCB fibres. The increase in strength in SFPCBs was primarily attributed to the strong bond formed between silica fume (SF), fibres, and the matrix phase of the concrete.
5. The addition of PCB fibres leads to a substantial enhancement of hardened concrete properties. Consequently, utilizing PCB waste in concrete production can result in the creation of concrete with superior characteristics, contributing to effective waste management and mitigating adverse environmental effects.

## Data Availability

The datasets generated and analysed during the current study are available from the corresponding author upon reasonable request.
